# Multiple COVID-19 Waves and Vaccination Effectiveness in the United States

**DOI:** 10.3390/ijerph19042282

**Published:** 2022-02-17

**Authors:** Lixin Lin, Yanji Zhao, Boqiang Chen, Daihai He

**Affiliations:** Department of Applied Mathematics, The Hong Kong Polytechnic University, Hong Kong, China; lixin.lin@connect.polyu.hk (L.L.); yan-ji.zhao@connect.polyu.hk (Y.Z.); boqiang.chen@connect.polyu.hk (B.C.)

**Keywords:** COVID-19, reinfection, breakthrough infection, vaccination effectiveness

## Abstract

(1) Background: The coronavirus 2019 (COVID-19) pandemic has caused multiple waves of cases and deaths in the United States (US). The wild strain, the Alpha variant (B.1.1.7) and the Delta variant (B.1.617.2) of severe acute respiratory syndrome coronavirus 2 (SARS-CoV-2) were the principal culprits behind these waves. To mitigate the pandemic, the vaccination campaign was started in January 2021. While the vaccine efficacy is less than 1, breakthrough infections were reported. This work aims to examine the effects of the vaccination across 50 US states and the District of Columbia. (2) Methods: Based on the classic Susceptible—Exposed—Infectious–Recovered (SEIR) model, we add a delay class between infectious and death, a death class and a vaccinated class. We compare two special cases of our new model to simulate the effects of the vaccination. The first case expounds the vaccinated individuals with full protection or not, compared to the second case where all vaccinated individuals have the same level of protection. (3) Results: Through fitting the two approaches to reported COVID-19 deaths in all 50 US states and the District of Columbia, we found that these two approaches are equivalent. We calculate that the death toll could be 1.67–3.33 fold in most states if the vaccine was not available. The median and mean infection fatality ratio are estimated to be approximately 0.6 and 0.7%. (4) Conclusions: The two approaches we compared were equivalent in evaluating the effectiveness of the vaccination campaign in the US. In addition, the effect of the vaccination campaign was significant, with a large number of deaths averted.

## 1. Introduction

The devastating COVID-19 pandemic, which caused by SARS-CoV-2, has provoked substantial infections and deaths in the US since its first case was confirmed on 21 January 2020 [1]. As of 24 December 2021, there have been 51,092,599 confirmed cases of COVID-19 with 803,744 deaths in the US reported to the WHO [2].

**Figure 1:**
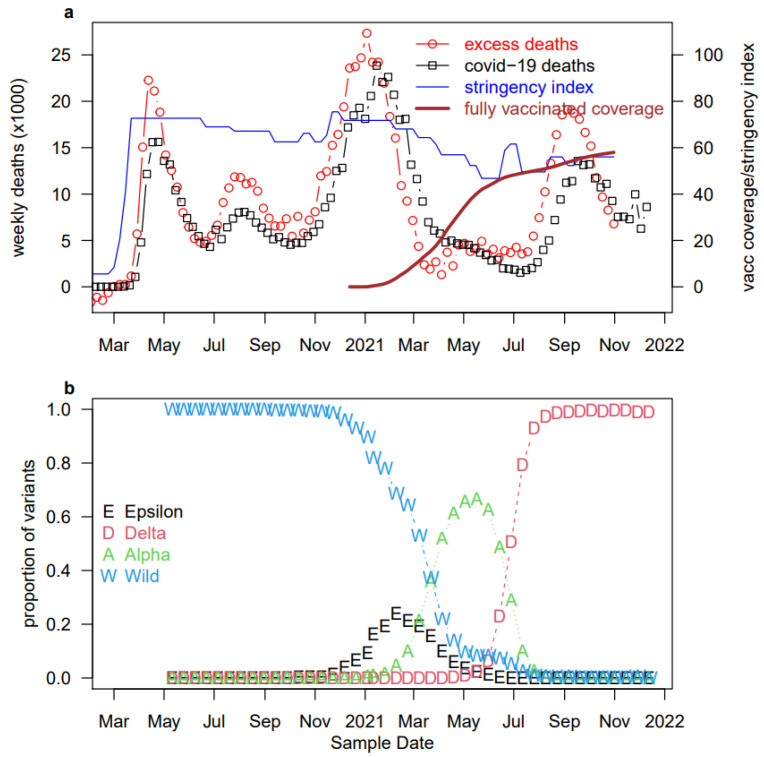
COVID-19 deaths [7,8], stringency index, vaccine coverage [9], and variant proportions [10,11,12,13] in the US. (**a**) Red empty circles (and black squares) represent the weekly excess deaths (and reported COVID-19 deaths) in the US. Excess deaths well match reported deaths, showing the high quality of death data. The red bold curve represents the percentage of fully vaccinated individuals, and the blue thin curve represents the stringency index which is a measure of control measure and population compliance. (**b**) Biweekly reported proportions of samples sequenced in the US. Overall, the Alpha variant replaced the wild strain, and subsequently, the Delta variant replaced the Alpha variant.

Overall, there were several waves of COVID-19 in the US, as shown in Figure 1. The initial stage was caused by the rapid spread of the wild strain, followed by the co-circulation of the wild strain and the Epsilon variant. The Epsilon variant was detected for the first time in California, USA, in July 2020 [3]. The Alpha variant, which was estimated to be 40–80% and more transmissible than the wild strain [4,5], replaced both the wild strain and the Epsilon variant and was finally replaced by the Delta variant. The Delta variant was first identified in India in December 2020 [6]. According to the Centers for Disease Control (CDC) tracking, the proportion of Delta variant infections among all samples sequenced in the US rose from nearly zero to nearly 100% during May 2020 and August 2021 due to the high infectivity of the variant. It was necessary and effective to take some interventions to mitigate the pandemic.

Iwasaki [14] detailed a case of SARS-CoV-2 reinfection in the US. However, the risk of reinfection in the US has been reported relatively rarely. The CDC COVID-19 Vaccine Breakthrough Case Investigations Team [15] counted 10,262 breakthrough infections reported to the CDC in the US, which accounted for 1% of the fully vaccinated population, from 1 January to 30 April 2021. Similarly, the degree of risk reduction for breakthrough infection versus initial COVID-19 infection is still controversial. To make conclusions about the risk of reinfection and breakthrough infection clearer, we conducted a systematic review of all relevant literatures published in PubMed through December 2021. As shown in Table 1 and Table 2, we concluded that the weighted average risk of reinfection and breakthrough infection decreased by 90.39 and 81.59%, respectively, compared with the risk of first-time infection.

To date, vaccine findings in reducing SARS-CoV-2 infection and mortality have not been uniform. Tartof et al. [16] investigated more than 3 million electronic health records between December 2020 and August 2021, and concluded that for fully vaccinated individuals, effectiveness against SARS-CoV-2 infection was 73% (95% confidence interval (CI): 72–74) and decreased from 88% (95% CI: 86–89) to 47% (95% CI: 43–51) from the first to the fifth month after full vaccination. Thompson et al. [17] highlighted that under real-world conditions, the mRNA vaccine was 90% effective in preventing SARS-CoV-2 infection in fully vaccinated individuals and up to 80% effective in partially vaccinated individuals. Through an event-study analysis, Li et al. [18] found that a phased approach to vaccination contributed to the reduction of the daily growth rate of COVID-19 cases in US. Blaiszik et al. [19] estimated from RT-PCR COVID-19 test data that the effect of Delta variant on vaccine efficacy was almost negligible.

Up to now, many works on the impact of COVID-19 and the efficacy of different interventions in the US have been published. Zou et al. [20] proposed a new epidemic model (SuIER) trained by machine learning algorithms to forecast the spread of COVID-19 in the US. They proposed that this model would provide accurate short-term (daily ahead) predictions of confirmed cases and deaths at the national and state levels, and the model predicted rapid increases of both in the long term. Reiner et al. [21] applied a deterministic SEIR compartmental framework to model possible COVID-19 infection trajectories and the effects of nonpharmaceutical interventions (NPIs) in the US at the state level from 22 September 2020 to 28 February 2021. Their findings indicated that the US may face a continued public health challenge from the COVID-19, and universal mask use could serve as a priority life-saving strategy in all states. They also suggested that new epidemics and resurgences are not inevitable.

As was predicted, there was a sustained resurgence in the transmission of COVID-19 that occurred in mid-2020 in the US. Monod et al. [22] incorporated the mobility data into a Bayesian contact-and-infection model. The results suggested that adults aged 20 to 49, especially those aged 35 to 49, contributed the most to resurgent COVID-19 epidemics. They also stated that additional interventions among adults aged 20 to 49, such as mass vaccination with transmission-blocking vaccines, could bring resurgent COVID-19 epidemics under control and avert deaths.

The US government has implemented many NPIs to reduce the rapid spread of COVID-19; however, the effectiveness of NPIs remains unclear. Singh et al. [23] used difference-in-differences methods to evaluate the impacts of implementing and lifting NPIs in US counties. Liu et al. [24] applied an established network-driven epidemic dynamic model to forecast the transmission of COVID-19 and the effectiveness of containment strategies. Courtemanche et al. [25] used an event study method to estimate the impact of social distance measures on the rate of growth of confirmed cases of COVID-19. The positive effect of NPIs on preventing the spread of COVID-19 was ascertained by all above papers.

Besides the NPIs, the mass vaccination with transmission-blocking vaccines is another effective intervention against the prevalence of COVID-19. Moghadas et al. [26] developed an agent-based model to analyze the impact of vaccinations on ongoing COVID-19 outbreaks in the US. This study showed that COVID-19 vaccines with 95% efficacy against this epidemic could amazingly reduce the attack rates across all age groups, the number of infections, and deaths. Using an age-structure model, Shim [27] found that vaccination program prevented more than 40% of deaths in South Korea. 

Many models have been proposed to analyze the impact of vaccination against COVID-19 [28]. However, previous models tend to be complicated and require many free parameters. While according to classic model selection theory, a useful model must have as few as possible free parameters [29,30]. Based on our previous work, we propose a model which incorporates vaccination. In particular, we note that there are two approaches to model the population level effect of vaccination. Through fitting our model to 51 states and regions in the US, we showed that the two approaches are equivalent. Furthermore, we calculate the lives saved due to vaccination across the US.

## 2. Methods

The classic SEIR model has been used by Song et al. [31,32] to study the impact of vaccination campaigns on COVID-19 epidemics in India and other countries in East Asia and Southeast Asia. In this article, we apply this model and evaluate the effects of vaccination in the US through two different approaches. 

$S,\;E,\;I,\;H,\;D,\;R,\;\mathrm{and}\;S_{V}$ denote the proportion of individuals who are susceptible, exposed, infectious, severe cases, dead, recovered, and fully vaccinated but weaker susceptible, respectively. η denotes the proportion of fully protected after vaccination (a proxy of the vaccination efficacy after the second dose). ψ denotes a reduced susceptibility of fully vaccinated individuals. Parameters σ, γ, κ denote the rate of exposure to infectiousness, loss of infectiousness, and severe cases leaving the severe stage, respectively. Parameters π and θ denote the ratios of infectious individuals entering H class, and individuals in H entering D class. The time-varying $\beta \left(t\right)$ is assumed to be an exponential cubic spline with the number of nodes $n_{\beta }$. In our model, we follow the valuation of parameters by Song et al. [31,32].

We assume $\pi =\theta ,{\;n}_{\beta }=9,\;\sigma =0.5/\mathrm{day},\;\gamma =0.33/\mathrm{day}\;\mathrm{and}\;\kappa =0.125/\mathrm{day}$. 

The model equations are:

(1) $$\dot{S}=-\beta SI-\tilde{v}S$$

(2) $$\dot{S_{V}}=\left(1-\eta \right)\tilde{v}S-\psi \beta S_{V}I$$

(3) $$\dot{E}=\beta I\left(S+\psi S_{V}\right)-\sigma E$$

(4) $$\dot{I}=\sigma E-\gamma I$$

(5) $$\dot{H}=\pi \gamma I-\kappa H$$

(6) $$\dot{D}=\theta \kappa H$$

(7) $$\dot{R}=\eta \tilde{v}S+\left(1-\pi \right)\gamma I+\left(1-\theta \right)\kappa H$$

The vaccine efficacy is:

(8) $$1-\left(1-\eta \right)\psi =\eta +\left(1-\eta \right)\left(1-\psi \right)$$

Namely, we move η proportion of vaccinated to R class, and the 1−η proportion to $S_{V}$ class, with a reduced relative susceptibility ψ as those susceptible in S. We argue that the effects of ψ and η are exchangeable. To confirm this, we consider two special cases to model the effect of vaccination: (i) ψ=1, η=0.85; (ii) ψ=0.15, η=0. 

The detailed fitting procedure of our model is well documented at https://kingaa.github.io/sbied/ (accessed on 5 December 2021).

## 3. Results

**Figure 2:**
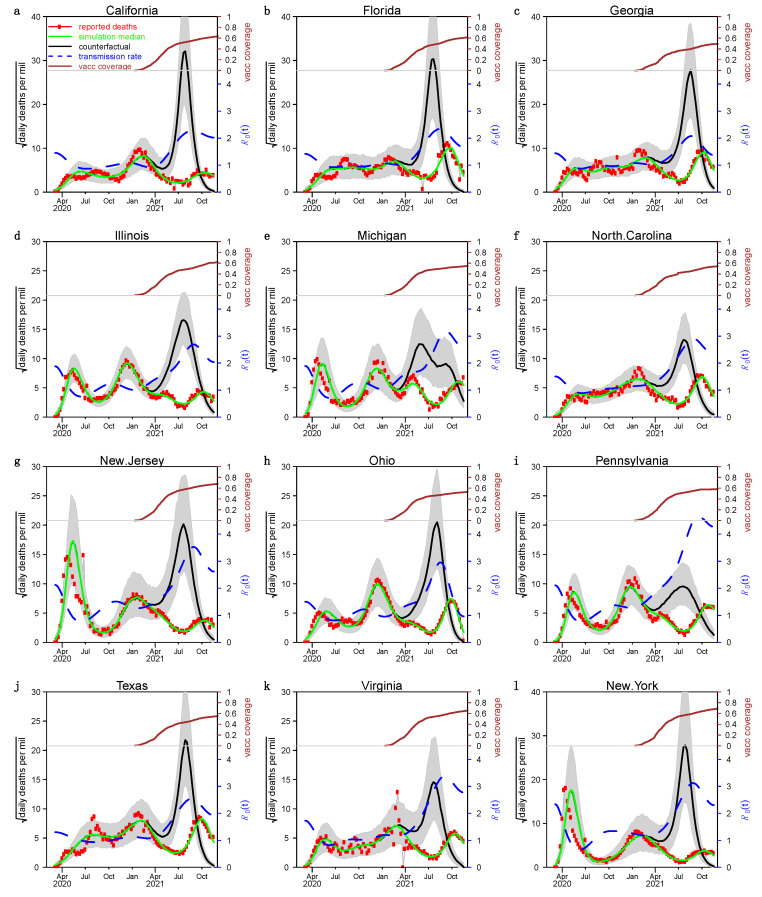
Fitting results under case 1. Panels (**a**–**l**) The model fit for the 12 most populous states in the US applying the first approach, respectively. Brown curves in the top of the panel show the vaccination (fully vaccinated) in each state. Red circles and green curves are observed and simulated (median, based on 1000 stochastic simulation runs) COVID-19 deaths. The black curve shows the simulated median under the scenario without vaccination. The blue dashed curve indicates the estimated transmission rate.

**Figure 3:**
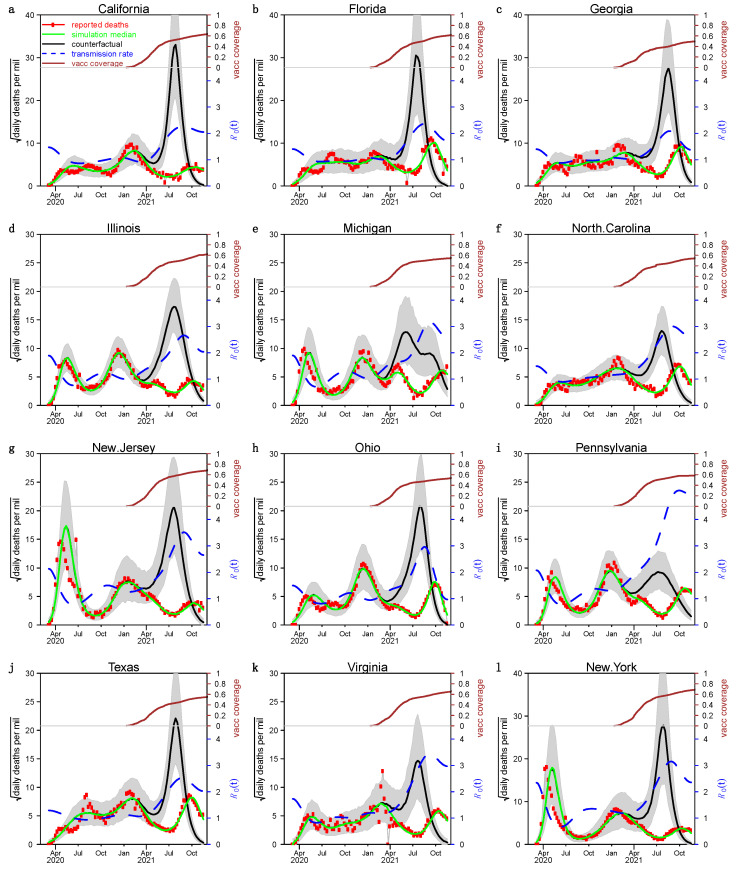
Fitting results under case 2. Panels (**a**–**l**) sowed the model fit for the 12 most populous states in the US applying the second approach, respectively. Others are the same as Figure 2.

We show the fitting and simulation results for the 12 states in the US with the largest number of people in Figure 2 and Figure 3, where the red circles, green curves, and black curves represent the number of reported severe acute respiratory infection (SARI) deaths, simulated deaths under the true scenario, and the counterfactual scenario of that without vaccination, respectively. The blue dashed line indicates the estimated transmission rate $\beta \left(t\right)/\gamma$. We estimate that the median IFR was about 0.6% and the mean was about 0.7%. The green curves were highly fitted to the number of reported deaths in each state, which indicated that our model-fitting results are ideal. The spacing of the green and black curves is highly significant in each state, suggesting that the vaccination campaign is very helpful in controlling the epidemic in the US.

**Figure 4:**
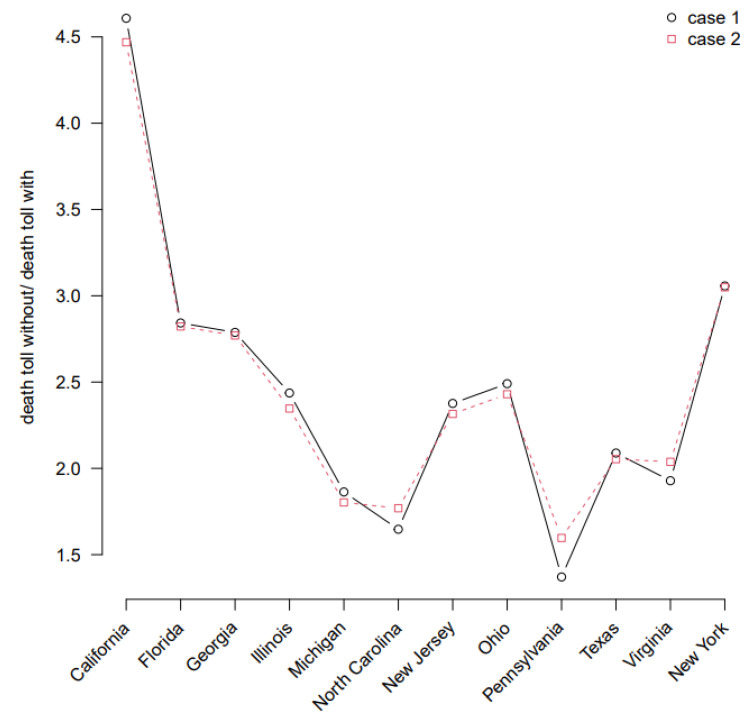
The ratio of estimated total deaths in the two scenarios for the 12 states in US with the highest population size fitted under the two approaches. Case 1 (ψ=1, η=0.85) and Case 2 (ψ=0.15, η=0) are the two special cases we listed in the Methods section.

As shown in Figure 4, by comparing the ratios of the estimated total number of deaths in the counterfactual scenario to the true scenario, we found that the results for both approaches are almost identical, and imply that vaccinated individual has a relative risk of infection of 1 − η (in the first approach) or ψ (in the second approach) compared to a non-vaccinated individual. 

We show the results for the other 38 US states and the District of Columbia in the Supplementary Materials. Figures S1 and S2 represent the fits for the second approach, and Figure S3 shows the ratio of total deaths predicted for the two scenarios corresponding to the two approaches. The conclusions are consistent with the 12 states we show in the main text. In addition, we visualize the reported daily number of cases and deaths for each state in the US in Figures S4 and S5.

Table 1 and Table 2 list the studies on the protective effects of infection-induced immunity and vaccine-induced immunity. By weighting, we derived a 90.39% reduction in the risk of reinfection and an 81.59% reduction in the risk of breakthrough infection compared to primary infection, respectively. Table 1 and Table 2 justified our model assumption on reinfection, namely reinfection in the short time interval (i.e., one year) before the invasion of Omicron variant is insignificant, thus reinfection was ignored. The breakthrough infection is modeled by allowing a proportion of vaccinated individuals staying the susceptible pool (in the first approach) or all vaccinated individuals moving to a susceptible pool with a reduced susceptibility.

## 4. Discussion

The US has experienced multiple waves of COVID-19 epidemics since February 2020. The US government has adopted approaches including NPIs and vaccination campaigns to mitigate and control the spread of the epidemic. To enable a clear analysis of the effectiveness of vaccination in the US, we combine the model of Song et al. [31,32] and propose two different approaches to modeling.

Hansen et al. [36] emphasized that the protective effect of prior infection against reinfection remained undiminished for more than 7 months based on a national dataset. However, Edridge et al. [47] highlighted that reinfection with the same seasonal coronavirus often occurred within 12 months of infection. Wangari et al. [48] proposed a model with a mechanism of reinfection transmission and emphasized that reinfected individuals would eventually lead to an increase in cumulative reported deaths. Coutinho et al. [49] estimated the transmissibility and reinfection of the P.1 (Gamma) variant based on an extended SEIR model and public health data, and derived a reinfection rate for P.1 variant. However, neither model [48,49] takes into account the possibility of reducing infectivity and pathogenicity of reinfected individuals, which may overestimate the severity of reinfection. Although there are certain cases of reinfection [14], the number is small with the under-reporting into consideration. In addition, previous works showed that prior infection provides nearly 90% protection against reinfection (for most variants prior to the Omicron variant). Therefore, in our model, we assume that the risk of reinfection is negligible for variants prior to the Omicron variant.

How to model the effect of vaccination is a difficult task in the COVID-19 modeling. We aim to show this problem in this aspect by comparing two approaches. To date, results from both clinical trials [50] and real-world data analysis [51] confirm the effectiveness of the vaccine (e.g., BNT162b2 vaccine) to be as high as 95% in the short term after the second dose. However, it is possible that the immunity produced by the vaccine will diminish over time. Mizrahi et al. [52] conducted a cohort study using data from Israel’s second-largest health organization and found that people who were vaccinated later had a significantly lower risk of contracting SARS-CoV-2 than those who were vaccinated earlier. In addition, the results of our literature review similarly suggest that studies with longer follow-up found a higher risk of breakthrough infection. In our model, we considered only individuals who received two doses of vaccine and ignored those who received only one dose. The population-level effect of the first dose should be taken over by that of the second dose [53]. Based on these considerations, we assume it is reasonable to take η to be in the range of 80–90%.

Unwin et al. [54] used a Bayesian hierarchical semi-mechanical framework that incorporated an autoregressive term capturing non-mobility-driven behavior to jointly model state-level epidemics in the US. Their model fitted the daily number of cases and deaths well for each state from February to June 2020. However, our model is much simpler, and we fitted 1 year and 8 months covering all waves of epidemics caused by COVID-19 in each state, compared with only 4 to 5 months in their work.

Moghadas et al. [26] used an agent-based SARS-CoV-2 transmission model parameterized by the US demographics and age-specific COVID-19 results, yielding an overall incidence reduction from 9.0 to 4.6% and a 69.3% reduction in the risk of death at 300 days post-vaccination. This is comparable to our findings, and we emphasize that the vaccination campaign has saved a large number of American lives, particularly between August and November 2021, by reducing the threat of the delta variant to the US. Although there was some variation across states, the number of deaths in most states could be 1.67–3.33 fold if the vaccine was not available, namely, most states reduced deaths by 40–70% through vaccination campaigns.

The strengths of our study are: first, we successfully fit a simple model with time-varying transmission and vaccination to multiple death waves in the US states from February 2020 to November 2021 at the state level; second, we consider two approaches to model vaccination and demonstrate that the two approaches are equivalent, which leads to the development of a unified model framework in assessing the effectiveness of vaccination in other places; third, our model is simple and has fewer free parameters than those of previous works modeling COVID-19 and vaccination. The drawbacks of our study, including the absence of reinfection, may lead to overestimating IFR. In addition, we only considered the second dose vaccination and ignored the impact of the first dose vaccination (which should be overtaken by the second dose quickly) [53]. A similar framework has been used in short-term forecasting [32] and modeling of the Omicron variant and other variants [55,56].

## 5. Conclusions

Based on the classic SEIR model, we proposed an SEIHDRS_V_ model and compared two different approaches to model the effects of vaccination. We concluded that the two approaches are equivalent. Either transferring fully vaccinated individuals to the recovered class in a certain proportion or transferring all of them to the class with reduced susceptibility to infection is feasible, which is useful for the development of a unified vaccination modeling framework and be used in other places. In addition, we emphasize that the vaccination campaign has saved a large number of American lives before the invasion of the Omicron variant.

## Figures and Tables

**Table 1:** Comparison of studies assessing the degree of reduction in risk of reinfection compared to primary infection (namely, the protective effects of infection-induced immunity).

Setting	Percent Reduction in Reinfection	Country	Sample Size	Follow-Up
Rovida et al. [33]	0.74	Italy	9610	6 months
Lumley et al. [34]	0.89	United Kingdom	12,541	7.3 months
Hall et al. [35]	0.841	England	25,661	9.3 months
Hansen et al. [36]	0.805	Denmark	525,339	10.1 months
Vitale et al. [37]	0.94	Italy	15,075	9.3 months
Hanrath et al. [38]	1	England	11,175	8.3 months
Pilz et al. [39]	0.91	Austria	8,900,480	9.3 months
Gallais et al. [40]	0.96	France	1309	12 months
Leidi et al. [41]	0.94	Switzerland	1496	8.2 months
Kohler et al. [42]	0.78	Switzerland	2712	7.9 months
weighted average	0.9039			

**Table 2:** Comparison of studies assessing the degree of reduction in risk of breakthrough infection compared to primary infection (namely, the protective effects of vaccine-induced immunity).

Setting	Percent Reduction in Breakthrough Infection	Country	Sample Size	Follow-Up
Rovida et al. [43]	0.86	Italy	4066	3.7 months
Santacatterina et al. [44]	0.91	USA	3975	3.9 months
Fowlkes et al. [45]	0.8	USA	7112	8 months
Naito et al. [46]	0.765	Japan	8749	6 months
weighted average	0.8159			

## Data Availability

Publicly available datasets were analyzed in this study. The death data originated from John Hopkins University can be found here: [https://github.com/CSSEGISandData/COVID-19] (accessed on 4 December 2021); the New York Times, found here: [https://github.com/nytimes/covid-19-data] (accessed on 4 December 2021); the SARS-CoV-2 variants data, found here: [https://ourworldindata.org/grapher/covid-variants-area] (accessed on 4 December 2021), [https://www.gisaid.org] (accessed on 4 December 2021), and the vaccination data, found here: [https://ourworldindata.org/covid-vaccinations] (accessed on 4 December 2021).

## Supplementary Materials

The following supporting information can be downloaded at: https://www.mdpi.com/article/10.3390/ijerph19042282/s1, Figure S1: Applying the second approach, the model fit for the other 19 US states and the District of Columbia. Others are the same as Figure 2; Figure S2: Applying the second approach, the model fit for the other 19 US states. Others are the same as Figure 2; Figure S3: Ratios of estimated total deaths in the two scenarios for the other 38 US states and the District of Columbia fitted under the two approaches; Figure S4: Daily number of cases from SARS-CoV-2 in all 50 US states and the District of Columbia from March 2020 to November 2021; Figure S5: Daily number of deaths from SARS-CoV-2 in all 50 US states and the District of Columbia from March 2020 to November 2021.
