# Supplementary material for: Multiple COVID-19 Waves and Vaccination Effectiveness in the United States
Source: Int J Environ Res Public Health. 2022 Feb 17;19(4):2282. doi: 10.3390/ijerph19042282 (PMC8871705; doi:10.3390/ijerph19042282)
Supplement: Supplementary file 1 [file ijerph-19-02282-s001.zip › ijerph-1549183-supplementary.pdf]

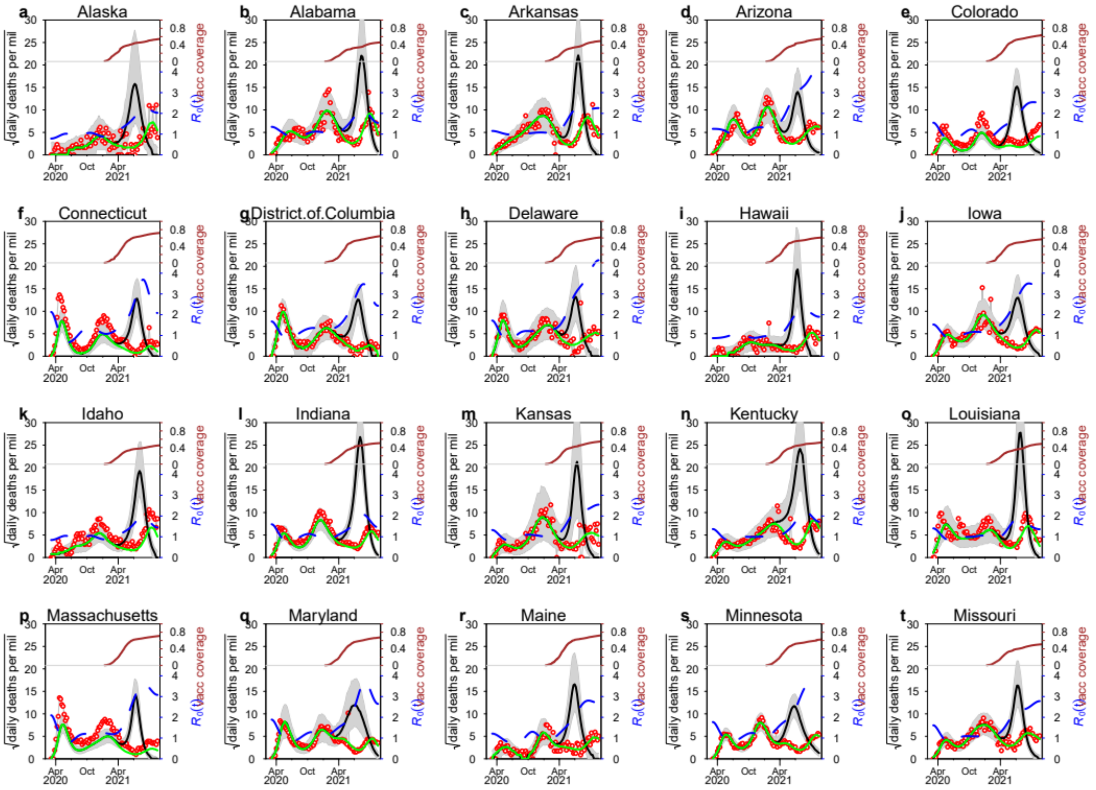

Figure S1. Applying the second approach, the model fit for the other 19 US states and the District of Columbia. Others are the same as Figure 2.

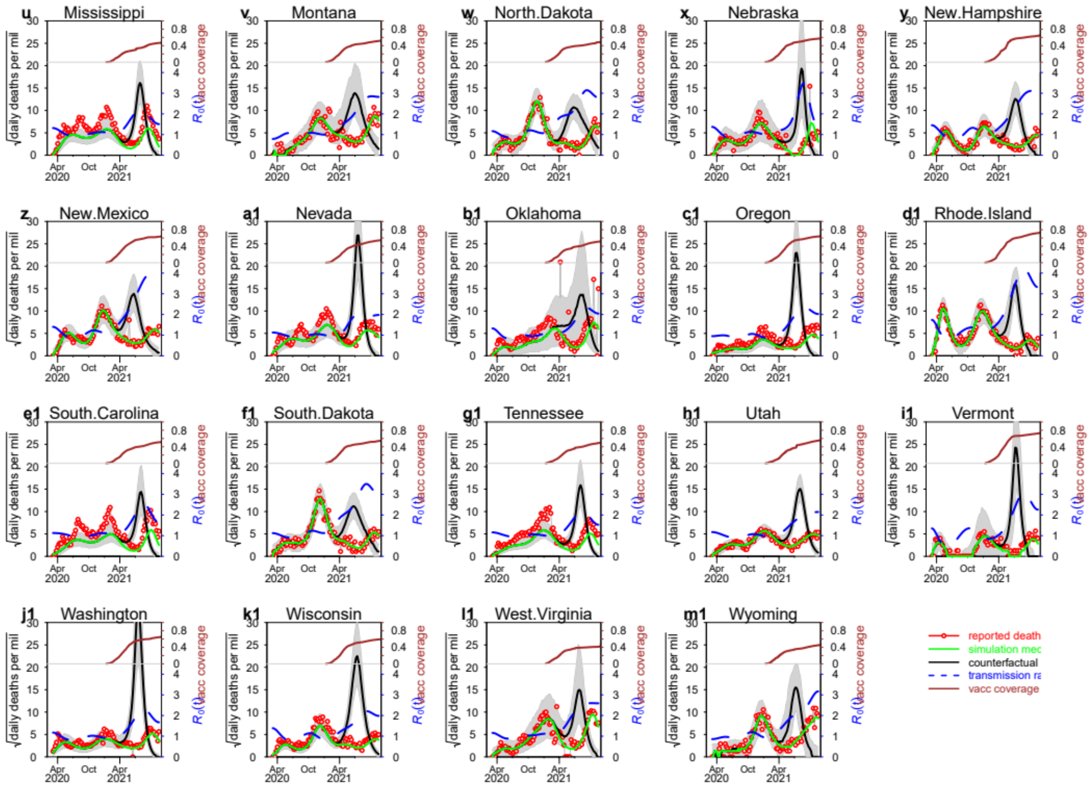

Figure S2. Applying the second approach, the model fit for the other 19 US states. Others are the same as Figure 2.

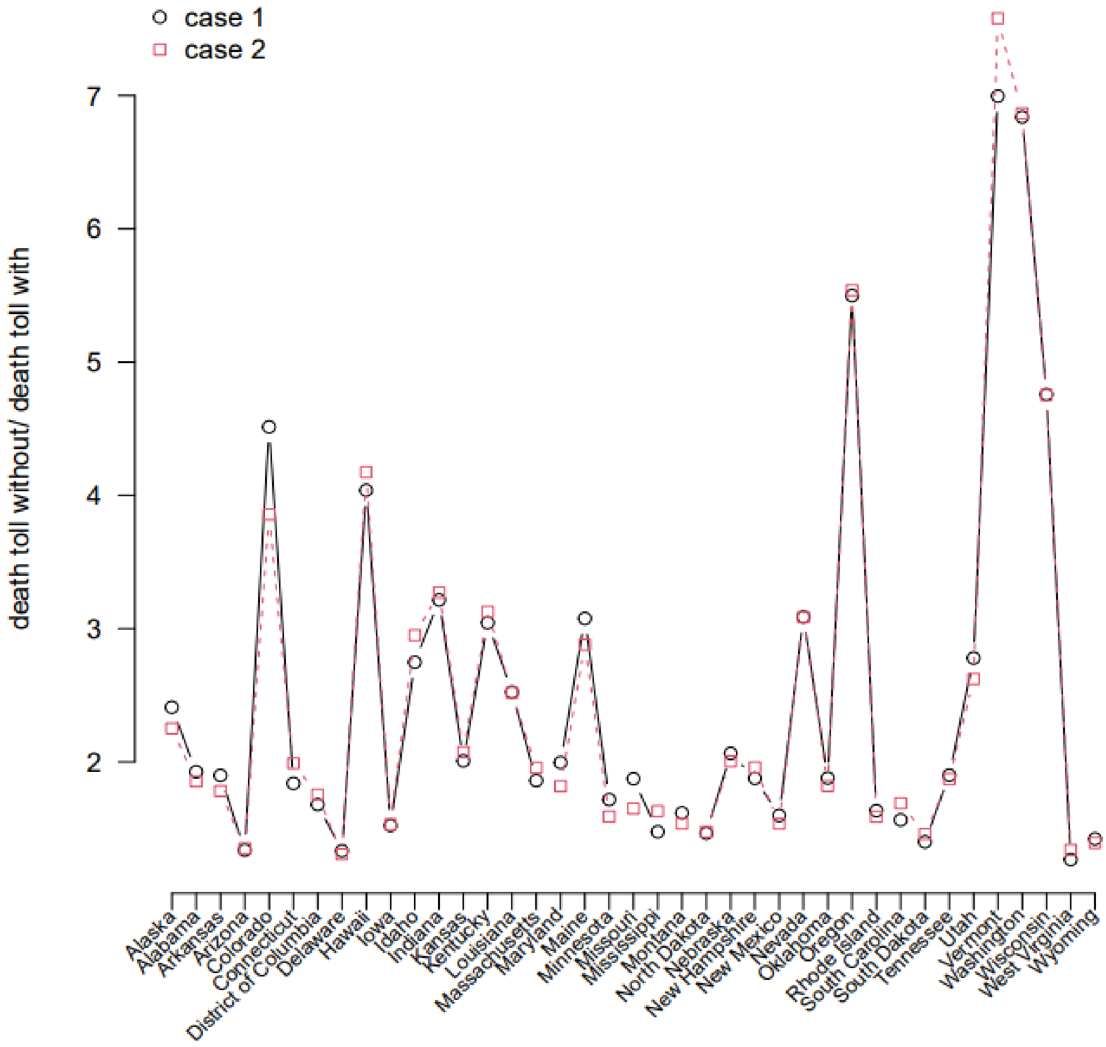

Figure S3. Ratios of estimated total deaths in the two scenarios for the other 38 US states and the District of Columbia fitted under the two approaches.

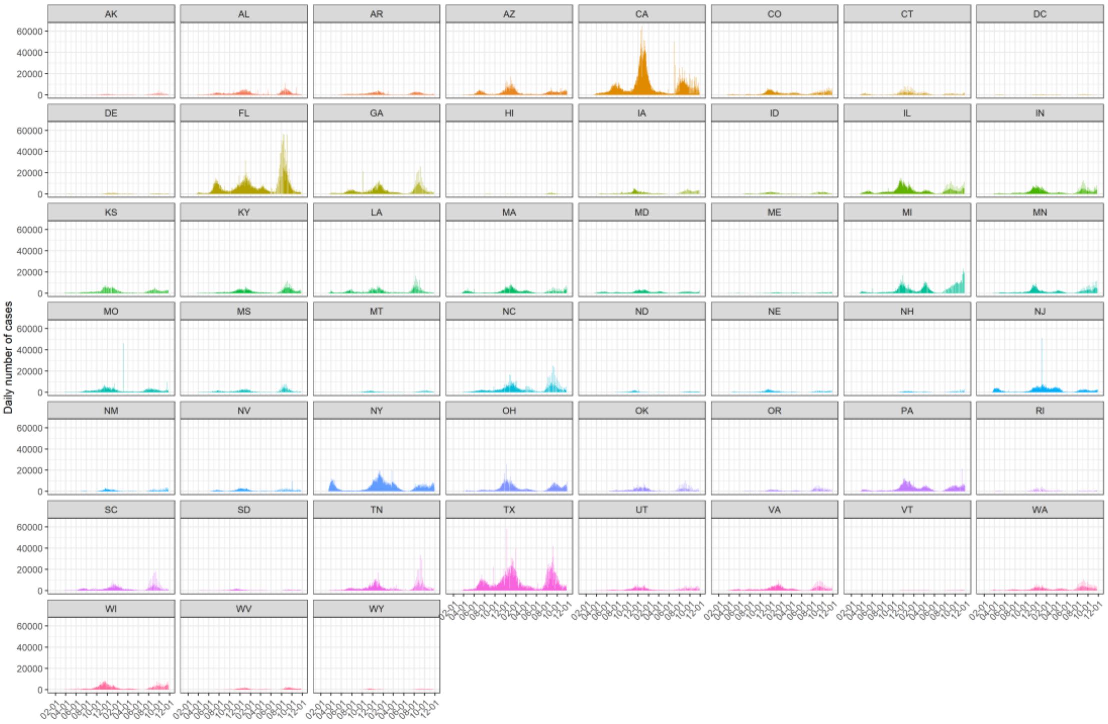

Figure S4. Daily number of cases from SARS-COV-2 in all 50 US states and the District of Columbia from March 2020 to November 2021.

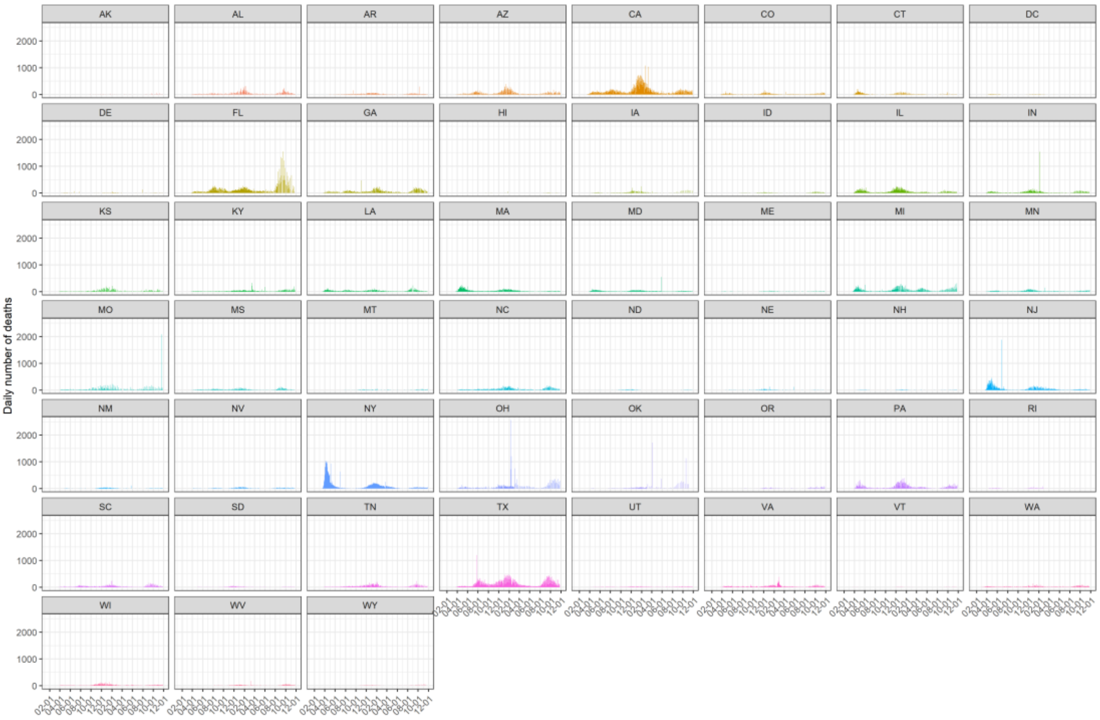

Figure S5. Daily number of deaths from SARS-COV-2 in all 50 US states and the District of Columbia from March 2020 to November 2021.
